# An Atypical Presentation of Non-Hodgkin’s Lymphoma

**DOI:** 10.7759/cureus.29052

**Published:** 2022-09-11

**Authors:** Joana Dias Antunes, Patrícia Almeida, Ivo Barreiro, Abílio Gonçalves

**Affiliations:** 1 Internal Medicine, Hospital Distrital da Figueira da Foz, Figueira da Foz, PRT

**Keywords:** spine, extranodal, large cell, non-hodgkin, lymphoma

## Abstract

Lymphomas result from the proliferation of malignant lymphocytes, which can affect lymph nodes, blood, and other organs. Primary involvement of the spine by haematological diseases is rare. Non-Hodgkin's lymphoma with an extranodal location most frequently involves the gastrointestinal tract and airways, affecting the bone, muscle, and nervous system.

We present a clinical case of an atypical form of non-Hodgkin's lymphoma. A 78-year-old woman was admitted to the hospital with complaints of pain in the lumbar region, hip, and left leg for the last month. Computed tomography of the lumbar spine revealed a mass of left paravertebral tissue with involvement from L3 to L5. Thoraco-abdominal CT-guided biopsy revealed diffuse large-cell non-Hodgkin B lymphoma. The remainder of the study did not show lymphatic involvement, so a diagnosis of primary extranodal large cell non-Hodgkin B lymphoma was made.

## Introduction

Lymphoma is a neoplasm of lymphoid tissue that results from the proliferation of malignant B and T lymphocytes. It usually originates in lymph nodes but can arise from any organ in the body. Extra-nodal lymphoma is defined by the involvement of organs and structures other than lymph nodes, such as the spleen, thymus, and pharyngeal lymphatic ring [1]. Differentiating disseminated disease from primary extra-nodal disease can be challenging.

Malignant non-Hodgkin's lymphoma (NHL) with extra-nodal location occurs in 10-20% of cases and most often involves the gastrointestinal tract (44%), upper airways (19%) and may reach the bone (8%), and central nervous system (5%) [1]. Spinal and spinal cord involvement is more frequently a late manifestation of systemic disease. Primary involvement of the spine by haematological diseases is rare, with a higher incidence in immunocompromised individuals [2].

Diffuse large B-cell lymphoma is the most frequent lymphoma subtype, representing 30-35% of all NHL [2]. It is also the lymphoma subtype that most frequently involves the cerebrospinal axis [2].

We describe a rare case of NHL whose presentation made the diagnosis challenging.

## Case presentation

**Figure 1:**
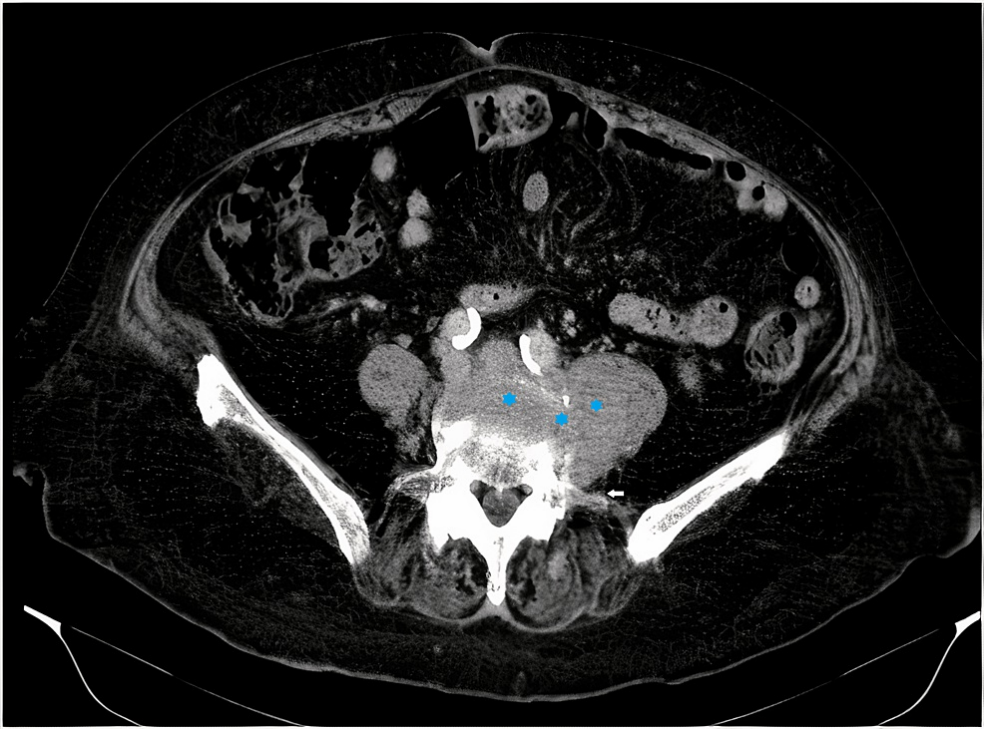
Computed tomography scan of the lumbar spine. Soft tissue mass that infiltrates the L4 vertebral body (stars), the pedicle, and left transverse apophysis (arrow), extending to the L3-L4 and L4-L5 intersomatic spaces and also intracanal extension.

**Figure 2:**
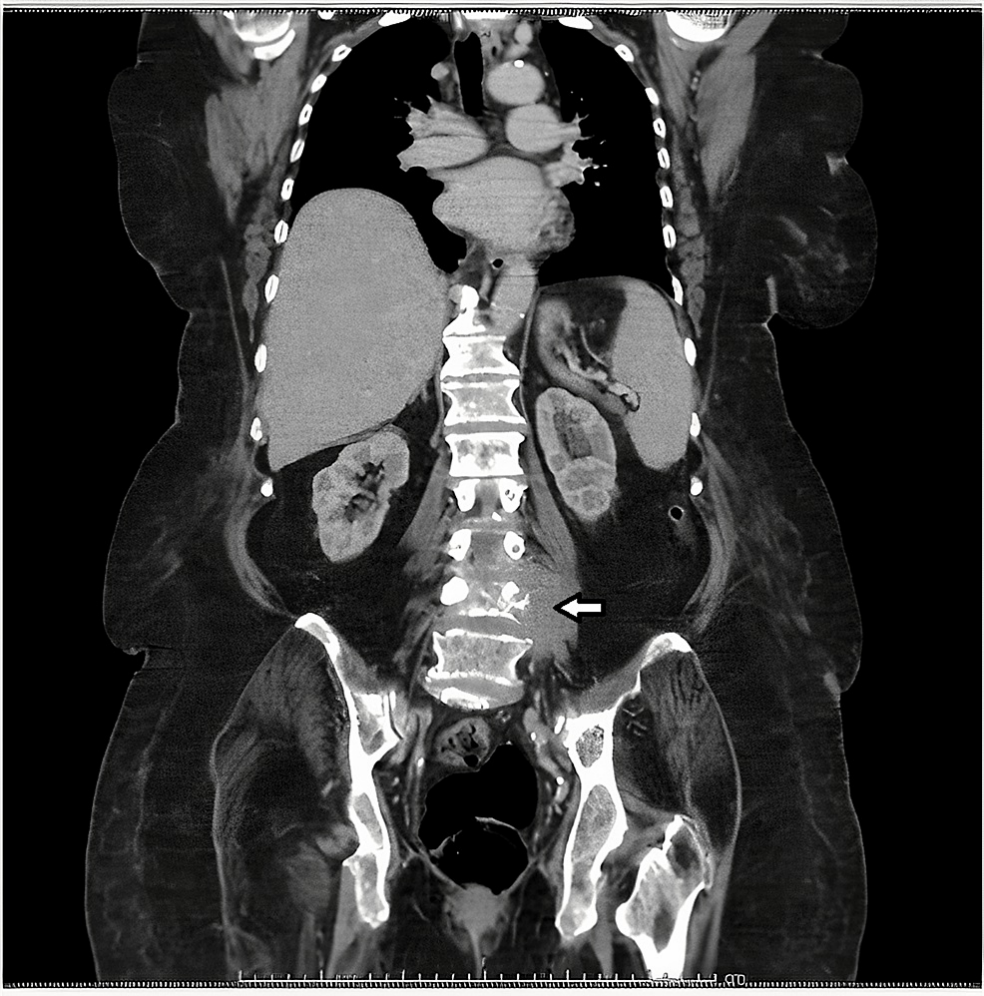
Thoraco-abdominopelvic computed tomography scan. Large mass component centered on the vertebral body of L4 and with greater left medial and anterior perivertebral expression (arrow), with signs of invasion of the psoas muscle and contacting the homolateral iliac vessels.

A 78-year-old female patient presented to the hospital with complaints of intense pain in the lumbar region, hip, and left leg, with 1 month of evolution, associated with asthenia and anorexia. She reported difficulty with ambulation in the 2 months prior. The patient had a computed tomography (CT) scan of the lumbar spine, performed outside the hospital, showing a left paravertebral soft tissue mass that infiltrated the vertebral body of L4, L3-L4, and L4-L5 intersomatic spaces, with intracanal extension and compromise of the roots at this level, suggestive of metastasis or an infectious lesion (Figure 1 and Figure 2). She was medicated with opioid analgesics since the onset of the condition, but with ineffective pain control.

She had type 2 diabetes mellitus, hypertension, hyperuricemia, and depressive syndrome and was medicated with sertraline 50mg once a day (QD), furosemide 40mg QD, pantoprazole 20mg QD, amlodipine 5mg QD, glargine insulin 16U QD, linagliptin 5mg QD, atorvastatin 20mg QD, allopurinol 100mg QD, gabapentin 200mg QD and tapentadol 100mg QD.

On physical examination, she was hemodynamically stable, subfebrile, and had a grade II/VI holosystolic heart murmur. She had a marked limitation of lower limb mobility. Strength was decreased (grade 3/5) in both legs and sensation was reduced. The knee-to-shin maneuver was normal. The knee-jerk reflex was depressed. The legs had hypotonia but the bulk was conserved.

The laboratory study on admission revealed leukocytes 10.8x10^3^/uL, hemoglobin 12.7 g/dL, platelets 282x10^3^/µL, erythrocyte sedimentation rate 70 mm/h, total protein 7 g/dL, albumin 3.5 g/dL, urea nitrogen 41.6 mg/ dL, creatinine 1.3 mg/dL, C-reactive protein 91 mg/L, and calcium 9.9 mg/dL. A protein gram was requested, which showed no monoclonal peak, immunoglobulin assay without alterations, and serum protein immunofixation without qualitative alterations.

The hypothesis of spondylodiscitis was possible, so the patient started antibiotic therapy with imipenem, after the collection of blood cultures. Collaboration was requested from the infectious diseases service, which considered the picture compatible with spondylodiscitis, so it was decided to extend antibiotic therapy to 6 weeks. Despite the negative blood cultures, there was a progressive clinical and analytical improvement (Table 1).

**Table 1:** Analytical evolution.

	Day 0	Day 5	Day 18
Hemoglobin (normal range 12.5-16 g/dL)	12.7 g/dL	12.3 g/dL	12 g/dL
Leukocytes (normal range 4-10.5x10^3^ uL)	10.8x10^3^/uL	8x10^3^/uL	5.9x10^3^/uL
Neutrophils	83%	76.1%	69.7%
Lymphocytes	10.7%	13.2%	18.4%
Platelets (normal range 150-450x10^3^ uL)	282x10^3^/µL	292x10^3^/µL	237x10^3^/µL
Calcium (normal range 8.4-9.7 mg/dL)	9.9 mg/dL		
Uric acid (normal range <5.7 mg/dL)		5 mg/dL	
Lactate dehydrogenase (normal range 240-480 U/L)	386 U/L	426 U/L	709 U/L
Creatinine (normal range 0.5-0.9 mg/dL)	1.3mg/dL	1mg/dL	0.7mg/dL
C-reactive protein (normal range <5mg/L)	91.03 mg/dL	59.5 mg/dL	12.45 mg/dL
Erythrocyte sedimentation rate (normal range <20mm/hour)	70 mm/h		34 mm/h

On day 5 of hospitalization, a CT-guided biopsy of the lesion was performed, uneventfully. Thoraco-abdominopelvic CT was performed which did not detect the presence of cervical, pulmonary or abdominopelvic adenopathies.

On day 20 of hospitalization, the anatomopathological report was available, which documented "infiltration by a non-Hodgkinian lymphoproliferative process, of lineage B - non-Hodgkin B lymphoma, probably high-grade/diffuse large cell."

In the subsequent complementary diagnostic study, bone marrow examination and phenotyping, bone biopsy, and urinary protein immunofixation did not show significant alterations and the investigation for hepatitis B and C and human immunodeficiency virus were negative. There was an increase in the level of beta-2 microglobulin (13,720 ug/L; normal between 800-2,200 ug/L). Positron emission tomography (PET) was ordered, which revealed a single lesion with moderate uptake of the radiopharmaceutical fluorodeoxyglucose-phosphorus 18 (FDG-18F), compatible with lymphoma with bone destruction at the level of L3 to L5. A definitive diagnosis of primary extra-nodal diffuse large B-cell lymphoma, stage I, was made.

A few days before the therapeutic decision consultation, there was a progressive clinical worsening of the patient, with loss of lower limb strength and multiple nosocomial infections. The rapid deterioration of her general condition led to her death a few days later, despite the initiation of therapy (steroids included).

## Discussion

The diagnosis of extranodal NHL is challenging, especially when there is the involvement of the spine and cerebrospinal axis, given its atypical presentation and the difficulty in obtaining a histological diagnosis. This location is very uncommon, corresponding to 1-2% of extra-nodal lymphomas [2]. It appears usually between the 5th and 6th decade of life, in males (ratio 1.6:1) [2].

The most common symptoms include pain localized in the spine, often due to a pathological fracture of the vertebra, and later neurological symptoms. In a small minority of patients, B symptoms appear [2,3]. Spinal cord compression typically presents with pain, paresthesias, or paresis of the extremities [4].

To confirm the primary extranodal lymphoma, a CT scan, MRI and PET-CT should be done [2]. Spinal MRI is one of the preferred imaging tests to assess the spinal cord and delineate the extent of tumor invasion at the level of the spinal space [5]. PET is an essential imaging exam for the study of this type of lesion, especially because large B-cell lymphoma has a high affinity for 18F-FDG [5]. Lesion biopsy is extremely useful for an early diagnosis and exclusion of other differential diagnoses. 

The treatment, although in the present case it was not possible to start, involves chemotherapy (CHOP or R-CHOP - rituximab, cyclophosphamide, doxorubicin, vincristine, and prednisolone) associated with intrathecal chemotherapy [6], radiotherapy, steroid therapy (in the case of spinal cord compression or patients at risk for central nervous system progression), bone marrow transplantation and surgery (for stabilization of the spine or in the case of refractory disease [2,5,6]). The therapeutic approach to primary vertebral NHL is not yet well established, given its rarity.

The prognosis of primary lymphoma of the cervical spine is unfavorable, with variable survival rates, with some studies reporting a first-year survival rate of 10% [2]. Poor prognostic factors include age over 50 years, more aggressive histological types, and neurological symptoms [7], factors that were present in this case.

## Conclusions

The importance of this case lies in the fact that it is an uncommon form of presentation of NHL, but it should be considered in the differential diagnosis of paraspinal masses or masses located in the spine. Early diagnosis and treatment are essential to improve the prognosis.

The disclosure of infrequent clinical cases can help other colleagues with the differential diagnosis of difficult-to-diagnose pathologies. Despite being rare, this case can become an useful reminder that rare pathologies should not be forgotten during the diagnostic process.

## References

[1] Moussaly E, Nazha B, Zaarour M, Atallah JP (2015). Primary non-Hodgkin’s lymphoma of the spine: a case report and literature review. World J Oncol.

[2] Popescu M, Popov V, Popescu G, Dobrea C, Sandu A, Grigorean VT, Strâmbu V (2012). Spinal involvement with spinal cord compression syndrome in hematological diseases. Rom J Morphol Embryol.

[3] Ciftdemir M, Kaya M, Selcuk E, Yalniz E (2016). Tumors of the spine. World J Orthop.

[4] D'Cruz J, Adeeb N, Von Burton G (2020). Diagnosis and management of intramedullary spinal cord lymphoma: a case illustration and review of literature. Interdisciplinary Neurosurgery.

[5] Hashi S, Goodwin CR, Ahmed AK, Sciubba DM (2018). Management of extranodal lymphoma of the spine: a study of 30 patients. CNS Oncol.

[6] Tilly H, Gomes da Silva M, Vitolo U (2015). Diffuse large B-cell lymphoma (DLBCL): ESMO clinical practice guidelines for diagnosis, treatment and follow-up. Ann Oncol.

[7] Yang W, Garzon-Muvdi T, Braileanu M (2017). Primary intramedullary spinal cord lymphoma: a population-based study. Neuro Oncol.

